# Effects of conditioning hops on drop jump and sprint performance: a randomized crossover pilot study in elite athletes

**DOI:** 10.1186/s13102-016-0027-z

**Published:** 2016-01-30

**Authors:** Jakob Kümmel, Julian Bergmann, Olaf Prieske, Andreas Kramer, Urs Granacher, Markus Gruber

**Affiliations:** Sensorimotor Performance Lab, Department of Sport Science, University of Konstanz, 78476 Konstanz, Germany; Division of Training and Movement Sciences, Faculty of Human Sciences, University of Potsdam, 14469 Potsdam, Germany

**Keywords:** Post-activation potentiation, Performance gains, Reactive movement, Plyometric exercise

## Abstract

**Background:**

It has previously been shown that conditioning activities consisting of repetitive hops have the potential to induce better drop jump (DJ) performance in recreationally active individuals. In the present pilot study, we investigated whether repetitive conditioning hops can also increase reactive jump and sprint performance in sprint-trained elite athletes competing at an international level.

**Methods:**

Jump and sprint performances of 5 athletes were randomly assessed under 2 conditions. The control condition (CON) comprised 8 DJs and 4 trials of 30-m sprints. The intervention condition (HOP) consisted of 10 maximal repetitive two-legged hops that were conducted 10 s prior to each single DJ and sprint trial. DJ performance was analyzed using a one-dimensional ground reaction force plate. Step length (SL), contact time (CT), and sprint time (ST) during the 30-m sprints were recorded using an opto-electronic measurement system.

**Results:**

Following the conditioning activity, DJ height and external DJ peak power were both significantly increased by 11 % compared to the control condition. All other variables did not show any significant differences between HOP and CON.

**Conclusions:**

In the present pilot study, we were able to demonstrate large improvements in DJ performance even in sprint-trained elite athletes following a conditioning activity consisting of maximal two-legged repetitive hops. This strengthens the hypothesis that plyometric conditioning exercises can induce performance enhancements in elite athletes that are even greater than those observed in recreationally active athletes.. In addition, it appears that the transfer of these effects to other stretch-shortening cycle activities is limited, as we did not observe any changes in sprint performance following the plyometric conditioning activity.

## Background

The muscle’s capability of generating high forces is dependent on its contractile history and can be acutely enhanced following voluntary contractions at maximal or near-maximal intensities [1]. These short-term enhancements can be observed on a behavioral and mechanistic level. In fact, acute conditioning-induced performance enhancements were reported for jumping and sprinting activities [2]. Most frequently, heavy resistance exercise protocols (e.g., squats with high loads) or maximal voluntary isometric contractions of the lower limb muscles have been used as conditioning activities to elicit athletes’ performance gains in jumping and sprinting tasks.

A few studies used plyometric exercise as a conditioning stimulus in order to increase countermovement jump (CMJ) [3–11], DJ [8, 12, 13], sprint [6, 11] and back squat performance [14]. It has been discussed that plyometrics have a high potential as a potentiating exercise to enhance athletes’ sport-specific performance due to similarities in their technical structure (e.g. explosive force or power) with sport-specific skills [10]. Terzis and colleagues for example have shown that 5 consecutive DJs significantly improved immediate following squat underhand front shot throwing distance by ~5 % [15], and 3 consecutive CMJs to elongate shot put distance by ~3 % [16]. Read and colleagues even found that CMJs increase subsequent golf club velocity of a golf swing [17]. In contrast, tuck jumps failed to improve the round kick force in karate athletes [10], and the 10 m and 20 m sprint performance of soccer players [6].

Those divergent findings related to the potentiating effect of plyometrics on subsequent performance enhancements have been attributed to the wide variety and diversity of methodological issues concerning the potentiating protocol, such as e.g. the level of activation during the conditioning [5], different resting periods [6, 18], and subsequent activities [9, 10]. These issues have a major effect on the concurrent incidence of potentiating mechanisms (e.g. activation of higher order motor units or enhanced contractile properties) and exhausting mechanisms (e.g. neuromuscular fatigue) on the subsequent performance [1].

Another point that has been discussed to affect the response to conditioning exercise is the training status and the strength level of the athletes [5]. There is evidence that human muscles with shorter twitch contraction times and a higher percentage of type II fibers exhibit a greater potentiating effect [19], proposing that strength and or power trained athletes whose muscles contain a greater type II muscle fiber cross sectional area benefit to a larger extent from the potentiating effect compared to their less active peers [2, 20]. A systematic review and meta-analysis on the potentiating effects on athletes’ performances revealed beneficial effects up to 6 % [21]. However two recently published studies found evidence for augmented lower body performance up to 12 % in recreationally active men, by means of 10 maximal repetitive reactive jumps (2 leg hops) prior to a subsequent DJ [12, 13]. It is unresolved whether athletes that are highly trained in strength and power activities can also benefit from this type of conditioning exercise. Further, there is limited information available whether those performance gains can be transferred to other stretch-shortening cycle movements. Therefore, the aim of this pilot study was to investigate whether a conditioning activity consisting of 10 repetitive hops can increases jump and sprint performance in highly strength and sprint-trained athletes who compete on an international level.

## Methods

A total number of 6 athletes participated in this study. However, one athlete could not participate in all measurements due to other reasons, thus he was treated as a drop out. Finally, 5 athletes completed the protocol of the present study (2 women and 3 men, means ± standard deviation (SD), age women: 23 ± 8 years, height women: 181 ± 3 cm, body mass women: 79 ± 8 kg; age men: 21 ± 2 years, height men 186 ± 14 cm, body mass men 99 ± 19 kg).

All participants were highly sprint-trained elite athletes competing on an international level. In addition, these athletes were well experienced in performing drop jumps since it was part of their daily training and testing routine. All of them gave their written informed consent to the experimental procedures. The study was conducted in accordance with the Declaration of Helsinki and approved by the local ethics committee of the University of Konstanz.

The athletes were tested on two separate days with at least one day of rest in between. DJ and sprint performance was assessed under two conditions in a random sequence on separate testing days, but always at the same time of day for each athlete to prevent any circadian effects. The PAP condition (HOP) afforded athletes to perform 10 repetitive reactive hops prior to each single DJ and each single 30 m sprint. The control condition (CON) included the same tests as in the PAP condition, however without any prior conditioning activity. The order of the sprint and DJ performance measurements was counterbalanced between all participants.

Prior to both experimental conditions (HOP, CON), the athletes performed a warm-up consisting of 25 heel rises, 45 s of submaximal repetitive two leg jumps (hops), and 15 squats as it was part of their general warm-up procedure prior to performance. Afterwards, they performed 3 DJs from a drop height of 46 cm to ensure consistent jumping technique in the subsequent tests. Participants had to jump barefoot, hands akimbo, and the heels did not touch the floor. In addition, they were instructed to conduct all hops and DJs with short ground contact times and maximal rebound jump height. Following this familiarization protocol, participants executed 8 DJs with a rest of 1 min between each DJ. During the HOP condition, the athletes executed 10 maximal hops 10 s prior to each jump (Bergmann interval) [12]. A break of 10 min was allowed between DJs and sprints. The 4 sprints were executed from an upright starting position on an indoor tartan track with the athletes wearing spiked shoes. Between each single sprint there was a resting period of 5 min. The athletes were asked to choose the sprint start on their own within the time frame of 10-20 s after the 10 hops were completed.

A force plate (Leonardo Mechanograph®, Novotec Medical, Pforzheim, Germany; sampling frequency 800 Hz) was used to record vertical ground reaction force and to quantify DJ performance. Contact times (CT_DJ_), rebound flight times (FT), peak forces (F_max_), and mechanical external concentric peak power relative to the individual athletes’ body mass (P_max_) were then calculated (Leonardo Mechanography Research Edition® software, Novotec Medical, Pforzheim, Germany). The performance index (PI) was calculated by dividing FT by CT_DJ_. Rebound jump height (JH) was determined by the following formula: $$ \mathrm{J}\mathrm{H}\kern0.5em =\kern0.5em \raisebox{1ex}{$1$}\!\left/ \!\raisebox{-1ex}{$8$}\right.\cdot \mathrm{g}\cdot {\mathrm{FT}}^2 $$ (g = gravitational constant).

An opto-electronic measurement system (OptoJump next®, MicroGate®, Bolzano, Italy) was used to quantify sprint performance over a distance of 30 m (spatial resolution: 0.01 m; sampling frequency: 1000 Hz). This system recorded the split sprint time over distances of 10 m, 20 m, and 30 m (ST_10_, ST_20_, and ST_30_). In addition, step length (SL) as well as contact times (CT_Sprint_) were measured during the first 10 meters of the sprint trials.

The mean values of 8 DJs and 4 sprints from each condition were used to compare performance variables between HOP and CON. Due to the limited number of study participants, differences between CON and HOP regarding JH and sprint performance (ST_10_, ST_20_, ST_30_, SL, and CT_Sprint_) were analysed using the non-parametric Wilcoxon signed-rank test for paired samples.

Additionally, dependent t-tests for paired samples were calculated to reveal intra-individual differences in the 8 drop jumps between CON and HOP. To do so, JH, CT_DJ_, P_max_, F_max_, and PI of the 8 jumps from both conditions were compared within each athlete. Group data are presented as mean values ± SD. Test-retest reliability was determined by means of a 2-way mixed model calculation for the intraclass correlation coefficient (ICC) on consistency [22] of each of the sprint and jump variables between the CON and HOP condition. Level of significance was set to p < 0.05.

## Results

**Fig. 1:**
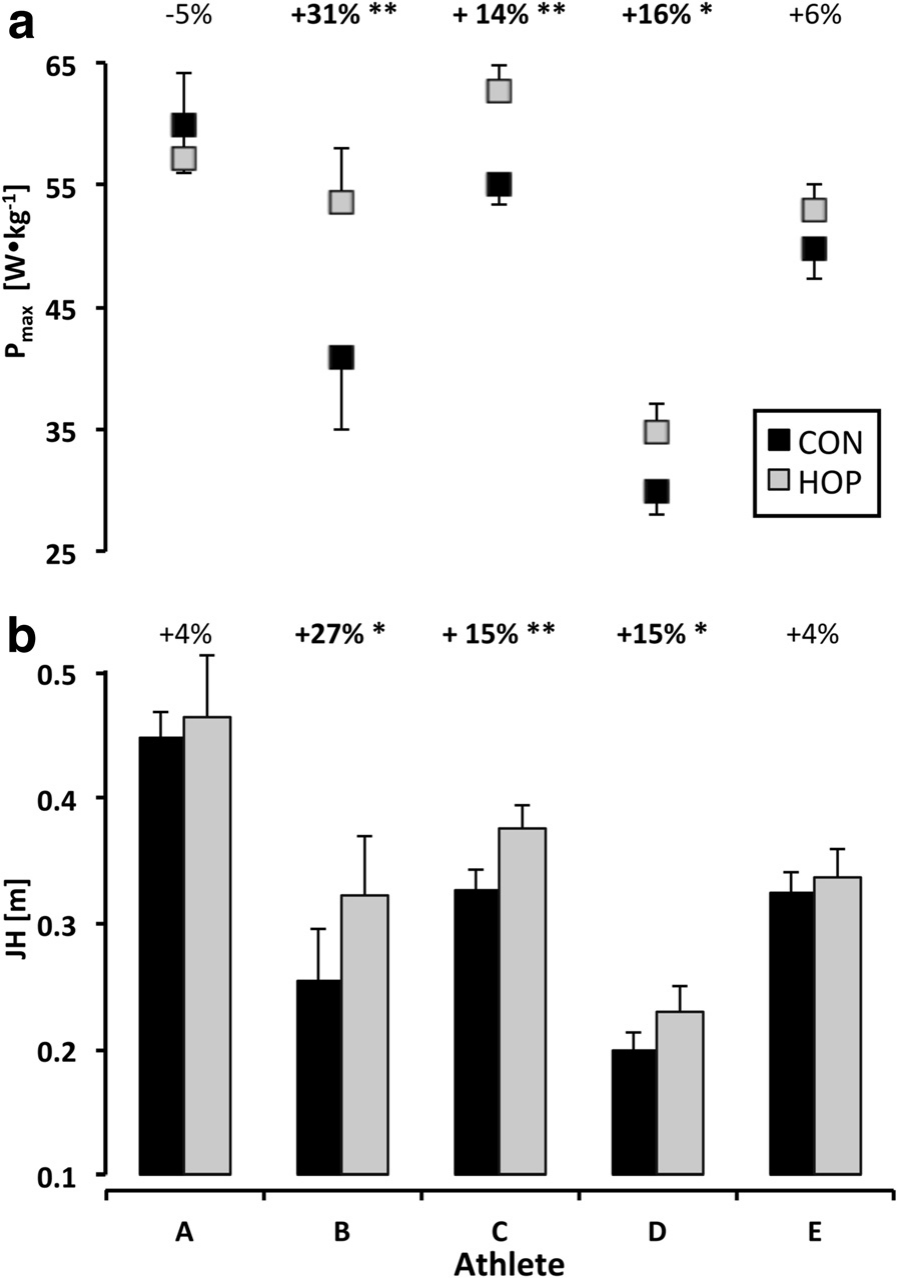
The individual mean (±SD) P_max_ (**a**) and JH (**b**) of 8 DJs under the control (CON; black squares/bars) and PAP (HOP; gray squares/bars) condition. P_max_ revealed higher values in the same 3 athletes that exhibited higher rebound jump heights in the HOP condition. In athlete E, P_max_ curtly failed to reach the level of significance (p = 0.05). Percentage differences related to the control condition are depicted above the bars of each single athlete. Asterix indicating significant within-subject differences (* *p* < 0.05 and ** *p* < 0.01) between CON and HOP. Nota bene, JH increased in each single athlete after the conditioning stimulus, however it was significant only in 3 out of 5

The Wilcoxon signed-rank test for paired samples revealed a significant increase in mean jump height (11 %; p < 0.05) in the HOP (0.35 m) compared to the CON (0.31 m) condition (ICC_JH_: 0.98, p < 0.01). The mean JH over the 8 DJs increased for each single athlete in the HOP compared to the CON condition (see Fig. 1). However, when analyzing the JH of each athlete’s jumps in the two conditions with dependent t-tests for paired samples, these differences were significant in 3 out of 5 athletes (B, C, D; ICC_Pmax_: 0.93, p < 0.05). The same 3 athletes reached a significantly higher P_max_ during the 8 DJs in the HOP compared to the CON condition (see Fig. 1). One of the two remaining participants (E) showed a tendency for a higher P_max_ value in the HOP compared to the CON condition (p = 0.051). Of note, athlete A showed a lower P_max_ value in the HOP condition compared to CON. The same athlete exhibited a significant longer CT_DJ_ in the HOP condition (13 %, p < 0.05), whereas athlete B had a shorter CT_DJ_ (10 %, p < 0.05, see Table 1; ICC_CT_: 0.88, p < 0.05). The PI increased individually between 6-24 % and this difference was significant for athletes B, C and D (ICC_PI_: 0.89, p < 0.05). In contrast to P_max_, no significant differences were found for F_max_ between the two experimental conditions in all analyzed participants (ICC_Fmax_: 0.94; p < 0.01).

**Table 1:** Individual mean values (±SD) of the parameters performance index (PI), contact time (CT_DJ_), and peak forces (F_max_) for 8 DJs under the CON and HOP condition

Athlete	PI		CT_DJ_ [s]		F_max_ [kN]	
	CON	PAP	CON	PAP	CON	PAP
A	3.1 ± 0.2	2.9 ± 0.4	0.194 ± 0.012	0.219 ± 0.022*	6.3 ± 0.6	6.9 ± 0.4
B	2.7 ± 0.3	3.4 ± 0.2**	0.169 ± 0.011	0.152 ± 0.013*	6.3 ± 0.4	6.3 ± 0.5
C	3.4 ± 0.1	3.7 ± 0.1**	0.150 ± 0.005	0.149 ± 0.007	6.5 ± 0.3	6.1 ± 0.4
D	2.1 ± 0.2	2.3 ± 0.1*	0.195 ± 0.014	0.186 ± 0.007	6.3 ± 0.3	6.0 ± 0.2
E	3.0 ± 0.1	3.2 ± 0.2	0.170 ± 0.008	0.164 ± 0.012	8.0 ± 0.3	8.0 ± 0.8
Mean (±SD)	2.9 ± 0.5	3.1 ± 0.5	0.176 ± 0.019	0.174 ± 0.029	6.7 ± 0.6	6.7 ± 0.6

An asterix indicates significant within-subject differences between the two experimental conditions (* *p* < 0.05 and ** *p* < 0.01). Group mean values (±SD) are presented in the bottom row

The mean values of ST_10_, ST_20_, and ST_30_ differed by less than 1 % (ICC_ST10_: 0.97; ICC_ST20_: 0.99; ICC_ST30_: 0.99, p < 0.01) and no significant differences were found between the two experimental conditions (Table 2). Similarly, step lengths (ICC_SL1_: 0.73, p = 0.12; ICC_SL2_: 0.91, p < 0.01) as well as contact times (ICC_CT1_: 0.93; ICC_CT1_: 0.86, p < 0.05) showed no significant changes between the HOP and CON conditions.

**Table 2:** Individual and group mean values (±SD) of the split sprint times over distances of 10 m, 20 m, and 30 m (ST_10_, ST_20_, and ST_30_) of the 4 30-m-sprints under the CON and HOP conditions

Athlete	ST_10_ [s]		ST_20_ [s]		ST_30_ [s]	
	CON	PAP	CON	PAP	CON	PAP
A	1.60	1.60	2.73	2.74	3.79	3.83
B	1.63	1.63	2.80	2.82	3.92	3.95
C	1.70	1.72	2.95	2.97	4.14	4.20
D	1.88	1.83	3.19	3.12	4.44	4.35
E	1.76	1.81	3.11	3.15	4.36	4.42
Mean (±SD)	1.71 ± 0.10	1.72 ± 0.10	2.96 ± 0.20	2.96 ± 0.18	4.13 ± 0.26	4.15 ± 0.25

## Discussion

In the present pilot study, we were able to demonstrate that 10 maximal repetitive hops can significantly increase performance in subsequent drop jumps in sprint-trained elite athletes competing on an international level. Thus, this study delivers further evidence for the high potential of plyometrics as a conditioning exercise to enhance lower limb performance, which is demonstrated by the significant increase in rebound jump height and peak power. However, the same conditioning activity failed to induce significant changes in 30-m sprint performance. More specifically, neither the 10 m and 20 m sprint time nor the kinematics of the first 10 m were affected by this type of conditioning activity. This result indicates a highly specific effect of the conditioning activity that fails to transfer into another complex sport specific task.

We used 10 maximal hops as conditioning with a rest of 10 s between pre-activation and performance assessment, as this protocol has been shown to induce remarkable performance increases in previous studies on recreational subjects [12, 13]. Reactive hops are characterized by high ground reaction forces, which have to be counteracted by the muscles of the lower limbs in a limited period of time in order to provide a basis for energy storage and recoil during the movement [23, 24]. These high forces were found to be sufficient to elucidate post-activation potentiation in the triceps surae muscle as well as increase DJ height [12]. This incorporates findings of previous studies that used conditioning exercise with a similar movement pattern and force characteristic to potentiate jump performances [4, 8]. Studies that used other types of plyometrics such as tuck jumps [5, 6, 10] or drop landings [25] either failed or reached only little potentiating effects on subsequent jump performance. Reasons for this are discussed to be the high load of the condition stimulus that masks the potentiating effect by concurrence of fatigue [5, 6], a failure in transferring the potentiating effect to higher power output [25], and a relatively low-force intensity being unable to induce a postactivation potentiation [6, 25]. We posit the latter point to be the major responsible reason for the equivocal findings of the abovementioned studies. The ground reaction forces that have been reported to appear during tuck jumps and depth jumps in athletes reach 3.6 times the athlete’s body mass [26]. Against this, there are indications in the literature for hops to reach values about 5 times their body mass [27, 28] and the athletes of the present study even reached an average peak ground reaction force of 7.5 times their body mass. Hence the results of the present study underpin reactive jumps to be the favorable plyometric conditioning activity when jump performance should be enhanced. This attempt of explanation for the divergent findings is further supported by the results of Masamoto et al. [14]. They have evidence for 2 DJs to increase the lower limb performance of a squat exercise after a rest of 30 s, whereas 3 tuck jumps had no impact on this exercise.

Contradicting this theory, Esformes at al. failed to show any potentiating effect of reactive jumps, including speed bounce and reactive hops, on subsequent CMJs [3]. A finding that was attributed to the low recruitment of muscle fibers during the conditioning activity. Besides the fact that the authors did not measure electromyographic activity during the conditioning exercise, this explanation deems to be insufficient since it is widely accepted that reactive movements have a high level of motor unit recruitment due to the pronounced contribution of the short-latency stretch-reflex component in the eccentric part of the movements [29–31]. We suppose that this is rather a matter of either less reactiveness in the execution of the vertical bounds or an exceeding resting period between the conditioning and performance assessment. Given that the muscles’ potentiated twitch peak torque is highest immediately following the conditioning activity and disappears in the proximate 5 min [12, 19], it is expected that performance increases might as well be largest immediately after a conditioning activity. This has been confirmed by two recently published original works, which provide evidence for the largest performance gains to occur immediately after (i.e. 10 s and 1 min, respectively) maximal repetitive hops [13, 32]. However, Lesinski et al. reported in their meta-analysis that the highest athletic performance increases occur after a rest interval of 6 - 10 min when moderate to heavy resistive exercises has been applied as conditioning exercise [21]. Bringing both points together, this might indicate a different time course of the net effect between potentiation and fatigue for a conditioning stimulus delivered by means of heavy resistance exercises versus a plyometric conditioning activity such as repetitive reactive hops. An exceeding resting period could be another reason for the missing effect of reactive conditioning exercise on improved jump performances in the study of Esformes and colleagues [3].

In line with previous findings in recreationally active participants [12], we found a significant increase in DJ performance of 11 % after the conditioning hops compared to CON. Since we used a similar conditioning and rest protocol as Bergmann et al. [12], it appears possible to argue that well-trained strength and sprint athletes competing on an international level can benefit from this kind of conditioning activity to a similar extent as compared to recreationally active individuals at least with regards to reactive movements. Due to the low sample size, we also analyzed individual changes in DJ performance after conditioning hops compared to control. Three out of 5 athletes (2 men, 1 w) showed significant improvements in JH following the conditioning activity. Increases ranged between 15 % and 27 % (Fig. 1). The two remaining athletes showed small changes in jump height (both +4 %), failing the level of significance. From these individual results we deduce that some reactively trained athletes can benefit remarkably from this kind of conditioning activity. In a study of Weber and colleagues, varying responses to a certain type of conditioning were attributed to the inhomogeneity of the participants [33]. Even though the authors did not intend to investigate individual responses to this kind of conditioning, they found individual performance gains from roughly +15 % to 0 % and even negative values were observed in two athletes. From studies using electrically evoked muscle twitches, it is known that the muscle fiber type distribution is an important factor determining the potentiating responses [19, 34]. Power-trained athletes should have a higher twitch response potentiation compared to endurance-trained athletes [35], as sprint-trained athletes exhibit a greater proportion of type two fibers in their muscles [36]. Due to the fact that all participants in the present study had a very high performance level with regard to lower limb explosive strength, which is demonstrated by the short sprint times (Table 2) and the high P_max_ values (Fig. 1), differences in fiber type composition appear to be an insufficient explanation for the inter-subject variability of the potentiation response. Apart from physiological factors, the demands of the testing task might have been different for each athlete. The DJ height was set to 46 cm for all participants, since the individual optimal DJ height could not be estimated in a prior session due to athletes’ time constraints. For the two athletes who had no significant improvements after conditioning, the drop height might have been higher than their individual optimal drop height [37, 38], thus reducing their mechanical power output [39] and possibly diminishing the potentiating effect [20].

Another interesting finding of this pilot study concerns changes in the biomechanical variables of the DJ movement. While for the conditioned DJs the JH, P_max_ and PI increased, whereby F_max_ remained unchanged. This is in accordance with findings from French et al., who observed that these changes were not associated with significant changes in ground contact time [40]. Hence, increased JH following the conditioning activity seems to be the result of an elevated force-time curve. Further analyses of the individual DJ performance revealed a significant increase of P_max_ in 3 athletes who had an augmented JH subsequent to the conditioning activity. Likewise, increases in external P_max_ have been reported by Chiu et al. in squat and CMJs following a conditioning activity of 5 squats at 90 % of the 1 repetition maximum [41]. An increased external concentric peak power is the result of a higher force during the concentric phase of the movement [42]. Since we were not able to detect any significant changes in F_max_ and CT, an elevated force level is a likely agent for the increased impulse during the concentric phase [40]. Even though some studies reported elevated F_max_ values in jumps following a conditioning activity [32, 40, 41], the present pilot study indicates that after hops, it is the overall shape of the ground reaction force rather than F_max_, which potentiates the DJ performance.

Several mechanisms are discussed to be responsible for enhanced performance following a conditioning activity. Intrinsic properties, such as changes in the muscle architecture or a higher Ca^2+^ sensitivity of the muscle fibers as well as recruitment of higher order motor units might cause the increased jump performance subsequent to the conditioning hops [1]. However, there is evidence that changes in neuronal output following the conditioning hops are not a likely mechanism responsible for the performance enhancements in subsequent DJs [12]. Several authors have proposed that the viscoelastic properties of the muscle-tendon unit might change subsequent to heavy resistance exercises [20] or plyometric conditioning [32]. In turn, this may result in a change in leg stiffness, allowing a higher contribution of passive tension to the overall force production during the concentric part of a stretch shortening cycle movement. In fact, it is known that there is an optimal leg stiffness value to maximize the external concentric peak power output during a DJ [39]. Therefore, it is possible that the significantly higher P_max_ in the athletes with considerably increased JH was due to changes in leg stiffness that brought it closer to the individual optimum, thus allowing a higher force contribution from the stretch-shortening cycle to the concentric propulsion phase of the DJ [20].

In contrast to other studies that used conditioning activities to enhance sprint performance [21], we were not able to demonstrate a decrease in sprint time (i.e., improved performance) in the present pilot study by means of a plyometric conditioning. Two studies reported a shorter sprint time of up to 3 % when using heavy loaded squats (60 % to 90 % of 1-RM) combined with resting intervals of 4 to 5 min as a conditioning stimulus [18, 43]. To our knowledge, only one study was able to show that plyometric exercises used as a conditioning activity can increase athletes’ sprint performance [44]. These authors used alternated-leg bounding either with or without an additional load (+10 % of the body mass). They reported up to 3 % shorter sprint times following weighted jumps compared to the control condition without prior conditioning activity, and smaller improvements with unloaded jumps. As the muscular load during alternate-leg bounds is higher compared to hops on two legs, it is possible that hops without additional load do not put sufficient load on the relevant muscles, which might be crucial for improved sprint performance. Nevertheless, the considerable performance gains in the DJs are in clear contrast to the lack of improvement in sprint performance. How can this high task specificity be explained? The results of training studies investigating the effects of different kinds of plyometrics on improvements in sprint performance suggest that plyometric training increases external leg power output during jumps, but has little or no effect on sprint performance [45]. This poor transfer was ascribed to contraction velocity specificity and differences in the movement between jumping and sprinting, requiring a different inter- and intramuscular coordination [45]. Therefore, a type of jump that is more similar to the movement during the acceleration phase of a sprint would probably yield better results when used as a conditioning activity. For example, CMJ height is related to the maximal sprint velocity [46, 47] and a good predictor of sprint performance, particularly over the first 30 m [48]. This may be due to the tendency that CMJs rely more on concentric muscle actions that generate energy, whereas reactive jumps with short ground contact times such as DJs and repetitive hops rely more on the stretch-shortening cycle and energy storage [49, 50]. Consequently, squat jumps and CMJs might be more suitable as performance-enhancing conditioning activities during the acceleration phase of a sprint than DJs or repetitive hops. And although the latter are closer to the movement in the constant speed phase of a sprint, they might not even be able to improve performance in this later phase, as the muscle activity in the preceding acceleration phase might interfere with any potential potentiating effects.

## Conclusion

Findings from this pilot study imply that in sprint-trained elite athletes 10 maximal repetitive hops can substantially increase performance in subsequent DJs. Compared to previous studies, the DJ results of the present study highlight the potential of a plyometric conditioning exercise to increase lower limb strength and power output, which can even can be effective in sprint trained elite-athletes. Equivocal findings related to plyometrics might arise from varying loads that have to be counteracted by the muscles during the conditioning exercises.

This type of conditioning activity did not affect sprint performance. We presume that the hops did not provide a sufficient conditioning stimulus for the muscle groups that primarily determine sprint performance.

The performance gains of the present study were most likely associated with an improved concentric propulsion phase of the DJs, making the stiffness of the muscle-tendon complex one likely candidate for the observed jump performance enhancement. Therefore, reactive hops might be more suitable as a warm-up routine prior to competitions in disciplines whose performances are rather determined by the stretch-shortening cycle, e.g. jump disciplines.

## Abbreviations

1-RM: one repetition maximum
CMJ: countermovement jump
CON: control condition
CT_sprint_ / CT_DJ_: ground contact time during sprinting/of a drop jump
DJ: drop jump
F_max_: peak ground reaction force in vertical direction
FT: rebound flight time
G: gravitational constant
HOP: hops conditions
ICC: intraclass correlation coefficient
JH: rebound jump height
P_max_: external concentric peak power
PI: performance index
SD: standard deviation
SL: step length
ST_10_/ ST_20_/ ST_30_: sprint time over a distance of 10 m, 20 m, 30 m
